# Audiological findings of family farmers exposed to pesticides

**DOI:** 10.1590/2317-1782/20232022108en

**Published:** 2023-09-01

**Authors:** Diolen Conceição Barros Lobato, Patrícia Arruda de Souza Alcarás, Denise Maria Vaz Romano França, Cláudia Giglio de Oliveira Gonçalves, Adrian Fuente, Adriana Bender Moreira de Lacerda

**Affiliations:** 1 Programa de Pós-graduação “Mestrado e Doutorado” em Distúrbios da Comunicação, Universidade Tuiuti do Paraná - UTP - Curitiba (PR), Brasil.; 2 Departamento de Educação, Universidade Estadual do Paraná - UNESPAR - Curitiba (PR), Brasil.; 3 École d´Orthophonie et d´Audiologie, Université de Montréal - UdeM - Montréal (Québec), Canadá.

**Keywords:** Agrochemicals, Hearing, Hearins Loss, Occupational Risks, Occupational Health

## Abstract

**Purpose:**

To analyze the possible differences among the hearing of farmers and their families when compared to the population not exposed to pesticides.

**Methods:**

Cross-sectional study with 70 smallholder family farmers (research group), with the mean age of 39.7 years, of both sexes and a mean of 23.7 years of exposure to pesticides. We included a control group with 71 participants of both sexes with the mean age of 39.5 years, not exposed to either noise or chemical substances, to compare the results. In stage 1, both groups were submitted to conventional and high-frequency audiometry, and acoustic immittance. In stage 2, only people with normal hearing were submitted to the evoked otoacoustic emissions and suppression effect on transient otoacoustic emissions.

**Results:**

Significant differences were observed between the groups in the conventional pure-tone and in the high-frequency audiometry, as well as in the acoustic reflex. The most affected frequencies in the conventional pure-tone audiometry ranged from 3 to 6 kHz and, in the high-frequency audiometry, from 9000 to 11200 Hz. As for the transient otoacoustic emissions, the worse suppression effect results were found in the research group.

**Conclusion:**

There were differences among the hearing of family farmers and the control group. The conventional auditory thresholds are related to the group, age and sex. Farming is associated with impairments in the basal region of the cochlea, absence of acoustic reflex, reduced signal-to-noise ratio of the transient otoacoustic emissions, and dysfunction in the olivocochlear efferents of the auditory system.

## INTRODUCTION

The family farmer^(^ 1 ^)^ is a worker who performs their duties in a rural area and, despite the existence of Occupational Health public policies, they are often unaware of their work environment risks to their health and their family’s, as well as the means to prevent harm or work-related effects.

Exposure to pesticides is one of the most noticeable risks among the family farmer’s work hazards, namely, chemical substances used in agriculture to change the composition of the flora or fauna, aiming to prevent harmful organisms from damaging the environment. However, its use not only alters the composition of the environment but also causes numerous detrimental effects on health^(^ 2-4 ^)^.

Regarding hearing health, studies indicate the presence of damages on the peripheral^(^ 3,5-7 ^)^ and central^(^ 3,8-11 ^)^ auditory system as well as hazardous effects on the vestibular system^(^ 6,12,13 ^)^. Impairments can be observed in the motility of the outer hair cells within the organ of Corti, progressing to the inner hair cells, auditory nerve, brainstem, and cortical regions, while in the vestibular auditory system, the author found lesions in the hair cells of the crista ampullaris in the saccule and the utricle^(^ 3 ^)^.

It is necessary to conduct an audiological monitoring and enroll the workers in Hearing Conservation Programs (HCP)^(^ 2,7,9,11,12 ^)^, since there is evidence that hearing impairments may be related to endogenous intoxication by pesticides^(^ 4,14 ^)^ and can be considered an early manifestation of chronic intoxication by said chemical agents^(^ 4,14 ^)^. Nevertheless, the studies that investigated the hearing of workers exposed to pesticides are composed mostly of male participants. Meanwhile, studies with female participants or in which the participants consisted of families of rural farmers (men, women, and children) exposed to pesticides are scarce to none.

In this context, the present research is justified by the sheer magnitude of families exposed to pesticides, given the relevance of Brazilian agriculture as a source of family income, especially in some municipalities in regions with few economic alternatives where agriculture plays an important role, in some cases being the only revenue and/or employment opportunity for certain groups of individuals.

Therefore, this study aimed to analyze the possible differences among the hearing of farmers and their families, composed of members of both sexes, owners of small agricultural establishments devoted to family farming with pesticides exposure in comparison with the not exposed population.

## METHOD

The present study was approved by the Human and Animal Research Ethics Committee of the Midwestern Paraná State University, COMEP/UNICENTRO, with the Official Letter No. 081/2011, cover No. 413146 and opinion No. 023/2011, dated October 17, 2011. It is a cross-sectional study developed in the state of Paraná (Brazil) with family farmers from rural cities. All participants were included in the study after signing the Informed Consent Form.

The assessments were carried out by the same professional but in three university clinics located in the state of Paraná-Brazil. The procedures were performed with the same criteria and care in all clinics where the data was collected, and all tests from the audiological evaluation were performed in the same period. The audiometry and otoacoustic emissions were performed in acoustic booths, measured annually according to the noise standards established by the ANSI S3.1^(^ 15 ^)^.

This study was conducted in two stages:

Stage 1

The participants were recruited by the Paraná State Health Department, actively searching for at least one case of agrochemical poisoning in the family, at the selected municipalities. Participants were invited by community health agents from the municipalities, by oral communication, in person, or by telephone.

The sample for Stage 1 consisted of two groups: research and control. The research group was composed by 70 participants, owners of small agricultural establishments devoted to family farming and their families, aged between 18 and 76 years (Mean = 39.7 years; SD = 13.4 years), presenting no cerumen or foreign body in the external auditory meatus, no previous chronic or otological diseases, with a normal middle ear, no occupational noise exposure (self-reported), and a mean of 23.7 years (SD=12.9 years) of agrochemical exposure, with a minimum exposure of one year and a maximum of 60 years, of which 26 (37%) were female and 44 (63%) male. The control group consisted of 71 participants from the database of Paraná University’s Audiology Services, with no history of exposure to noise and chemical substances (self-reported), aged between 18 and 67 years (Mean = 39.5 years), with 27 (38%) females and 44 (62%) males.

The research group reported having contact with various types of pesticides, such as glyphosate (80%), dinitroaniline (53%), organophosphate (51%), pyrethroid (49%), neonicotinoid (46%), dithiocarbamate (43%), carbamate (17%) and organochlorine (3%). The handling of pesticides involves the preparation of the syrup (57%), application (85%), and washing of the material (67%). 70% of this group reported applying pesticides with a backpack pump, 8% with a spray and 15% reported not using any type of equipment. During the application, 41% reported wearing boots/shoes, 31% shirts, 29% gloves, 27% mask with filter, 25% pants, 21% overalls, 19% goggles, 12% head protection, 4% mask without filter, and 4% disposable clothing.

The participants underwent a meatoscopy with a Mikatos otoscope, assessing the external acoustic meatus to observe the presence or absence of a foreign body, which would prevent the proper performance of the audiological evaluation. Subsequently, a pure-tone audiometry was performed in search of the thresholds in the frequencies of 250, 500, 1000, 2000, 3000, 4000, 6000, and 8000 Hertz (Hz) by air as well as 500, 1000, 2000, 3000, and 4000 Hz by bone, when the airway was altered. Thresholds within the range of up to 25 dBHL were considered normal for all frequencies, according to Appendix II of NR - 7^(^ 16 ^)^. The audiometry was performed with two audiometers: one from Otometrics, using the Madsen Itera II model with the TDH-39 supra-aural headphones, and another from Damplex, the DA 65 model with the TDH-39 supra-aural headphones, duly calibrated. High-frequency audiometry was performed with an Otometrics audiometer, using the Madsen Itera II model with HDA-200 supra-aural headphones and dBHL stimulus, evaluating the frequencies by air conduction of 9000, 10000, 11200, 12500, 14000, and 16000Hz, comparing the results were with the control group^(^ 17 ^)^.

Two devices were also used for the acoustic immittance measurements: one from Interacoustics, model AT22, and another from Audiotest, model 425, duly calibrated. The acoustic immittance measurements were: tympanometry and the investigation of the acoustic reflex, both contralateral and ipsilateral, at the frequencies of 500, 1000, 2000, and 4000 Hz. The tympanometry results obtained in static compliance were considered normal between 0.3 ml and 1.3 ml, with peak middle ear pressure between -100 daPa. Values below 0.3 and above 1.3 were considered abnormal, as well as middle ear pressures below -100 daPa and above +100 daPa. The acoustic reflex was considered present when triggered at the maximum intensity allowed by the equipment (120 dBHL in the contralateral afferent and 110 dBSPL in the ipsilateral afferent), and absent when not triggered at the maximum intensity allowed by the equipment^(^ 18 ^)^.

Stage 2

Were included only the participants who presented hearing thresholds within the normal range^(^ 16 ^)^ or with a hearing loss ≤ 40 dBHL, according to a mean of 500, 1000, and 2000 Hz^(^ 18 ^)^ or at an isolated frequency (3000, 4000, 6000, or 8000Hz) and with type A tympanometric curve.

The research group (n = 24) consisted of participants aged between 24 and 61 years (Mean = 36.8 years), being 14 females and 10 males, with a mean risk exposure time of 20.7 years (minimum exposure two years and maximum 46 years). The control group (n = 24) was paired with the research group, considering age as well as sex, and consisted of participants without exposure to noise and chemical products, with a mean age of 34.7 years, ranging from 21 and 53 years old, being 14 females and 10 males.

Participants in both groups were submitted to the Transient Stimulus Otoacoustic Emissions (T-OAE), the Distortion-Product Otoacoustic Emissions (DP-OAE), and the T-OAE Suppression Effect.

For the investigation of evoked otoacoustic emissions (T-TOAE, DP-OAE, and T-OAE Suppression Effect) was used the computer program ILO-V6 - Otodynamics Analyzer, coupled to an HP notebook. This equipment has a probe (ILO Type OAE Probe) whose function is to release the sound stimulus, as well as to receive and measure the responses in the external auditory canal. This probe is connected to two channels and to an interface attached to the notebook.

In the T-OAE exam, the following protocol was used: click stimulus at an intensity of 80 dB SPL, 400 scans. The response pattern’s general reproducibility was greater than 50%, probe stability greater than 70% and response level greater than the noise, with a signal-to-noise ratio ≥ 3dB SPL in at least three or more consecutive frequencies^(^ 19 ^)^.

The DP-OAE assessment was performed with two primary tones (f1 and f2), with f1 being the lowest frequency and f2 the highest frequency. The distance between the two frequencies (f1 and f2) was obtained in a ratio of f2/f1 = 1.22, to obtain the ideal distortion product in the “General Diagnosis” mode, using the intensity level of L1= 65 dB SPL and L2= 55 dB SPL. The distortion product response was obtained at 2f1-f2 and reported at the f2 frequency as 1001, 1587, 2020, 3174, 4004, 6348, and 7996 Hz. Probe stability greater than 70% and a signal-to-noise ratio ≥ 6 dB SPL per specific frequency were considered^(^ 19 ^)^.

To analyze the T-OAE suppression effect, the participants who presented transient otoacoustic emissions, with a signal/noise ratio (S/N) equal to or greater than 3 dB SPL in three consecutive frequencies and at least one ear, underwent the suppression effect test. Without repositioning the probe, the T-OAE suppression was recorded according to the following protocol: click stimulus, linear, with stimulus intensity at 60 dB SPL (± 5) and contralateral white noise at 60 dB SPL (± 5). An average of 500 scans were performed, 250 scans in the absence of contralateral noise and 250 in the presence of contralateral noise. The suppression was assessed in each ear (right and left). The suppression effect was evaluated observing the general response level in the presence of contralateral noise when compared with the general response level without the contralateral noise, using two types of responses as reference: Present suppression, when there is a reduction in the general response level of emissions in the presence of contralateral noise (values greater than or equal to 1.0), and Absent suppression, when there is no reduction in the general response level of emissions in the presence of contralateral noise (values less than 1.0)^(^ 20 ^)^.

The statistical analysis of this research was performed using descriptive and inferential methods. The descriptive methods (absolute and relative frequency tables, with mean, minimum, maximum and standard deviation) used to characterize family farmers were based on the following variables: age, sex and time of agrochemical exposure, as well as signs, symptoms, risks, conventional results, and high-frequency audiometry. The inferential methods were: Student's t-test (to compare the amplitudes and S/N ratio in the T-OAE and DP-OAE, in addition to the suppression effect), Spearman Correlation Coefficient (to assess the correlation between the audiological tests: pure-tone audiometry, high-frequency audiometry, T-OAE, DP-OAE and T-OAE suppression effect, in addition to laboratory tests), Man Whitney Test (to compare the hearing thresholds of family farmers in pure-tone audiometry and high-frequency audiometry), Multiple Linear Regression Model (Dependent variable: audiometric thresholds; independent variables: group, age and sex), and G test (acoustic reflex assessment). All tests considered the significance level of 5% (0.05), that is, there is statistical significance when the p-value is less than or equal to 0.05.

## RESULTS

Tables 1, 2, and 3 show the results of the auditory threshold characterization obtained through the conventional tone threshold audiometry and high frequencies of the research and control groups.

**Table 1:** Characterization and comparison of conventional pure-tone audiometry in the research group (RG) and control group (CG), obtained in the right (RE) and left (LE) ears (N = 141)

**FREQ**	**EAR**	**GROUP**	**MEAN**	**MEDIAN**	**SD**	**MIN**	**MAX**	**P-VALUE**
**250Hz**	RE	RG	14.79	15	8.741	5	60	* **0.000**
		CG	10.00	10	4.053	0	5	
	LE	RG	13.57	15	8.269	-5	50	*0.000
		CG	8.87	0	3.891	0	20	
**500Hz**	RE	RG	13.57	10	6.818	5	40	*0.000
		CG	9.30	10	4.728	0	20	
	LE	RG	12.79	10	7.403	0	45	*0.001
		CG	8.59	10	4.719	0	15	
**1000Hz**	RE	RG	11.43	10	7.231	0	40	0.069
		CG	9.00	10	5.664	0	20	
	LE	RG	11.36	10	8.678	0	50	0.206
		CG	8.95	10	5.664	0	25	
**2000Hz**	RE	RG	13.07	10	12.047	0	60	*0.022
		CG	8.50	10	6.740	0	20	
	LE	RG	12.71	10	13.875	-5	60	0.418
		CG	8.30	10	6.900	0	35	
**3000Hz**	RE	RG	16.07	10	16.127	-5	65	*0.018
		CG	9.37	10	7.316	0	25	
	LE	RG	16.57	10	18.209	-5	70	0.074
		CG	10.21	10	8.760	0	40	
**4000Hz**	RE	RG	18.93	15	17.464	-5	75	*0.017
		CG	11.55	10	8.088	0	35	
	LE	RG	19.93	15	17.703	-5	70	*0.001
		CG	11.70	10	10.452	0	55	
**6000Hz**	RE	RG	22.93	20	16.539	5	85	*0.000
		CG	13.00	15	7.995	0	40	
	LE	RG	24.00	20	17.664	-5	90	*0.000
		CG	13.38	10	10.750	0	50	
**8000Hz**	RE	RG	17.93	15	16.495	0	90	0.104
		CG	13.96	10	9.949	0	40	
	LE	RG	20.43	15	18.013	-5	75	*0.016
		CG	14.44	10	14.231	0	65	

Mann-Whitney test

* p < 0.05 (significant p-value)

**Caption:** EAR= laterality of the ear, Hz = Hertz, FREQ = frequency, SD = standard deviation, MIN = minimum, MAX = maximum

**Table 2:** Analysis of the multiple linear regression model concerning the comparison of the conventional auditory thresholds according to groups (research and control), age, and sex (N = 141)

	EAR	**250 Hz**	**500 Hz**	**1 kHz**	**2 kHz**	**3 kHz**	**4 kHz**	**6 kHz**	**8 kHz**
**Group**	RE	*0.000	*0.000	*0.015	*0.002	*0.000	*0.000	*0.000	* **0.007**
	LE	*0.000	*0.000	*0.022	*0.028	*0.002	*0.000	*0.000	*0.007
**Age**	RE	*0.000	*0.000	*0.000	*0.000	*0.000	*0.000	*0.000	*0.000
	LE	*0.000	*0.000	*0.000	*0.000	*0.000	*0.000	*0.000	*0.000
**Sex**	RE	*0.010	>0.05	>0.05	>0.05	*0.005	*0.009	>0.05	*0.010
	LE	*0.000	>0.05	>0.05	>0.05	*0.002	*0.001	>0.05	*0.006

Multiple linear regression model

* p < 0.05 (significant p-value)

**Caption:** EAR=laterality of the ear, Hz = Hertz, RE = right ear, LE = left ear.

**Table 3:** Characterization and comparison of pure-tone audiometry at high frequencies in the research group (GP) and control group (CG), obtained in the right (RE) and left (LE) ears (N = 141)

**FREQ**	**EAR**	**GROUP**	**MEAN**	**MEDIAN**	**SD**	**MIN**	**MAX**	**P-VALUE**
9.000 Hz	RE	RG	24.00	15	17.565	0	90	* **0.006**
		CG	19.29	15	19.821	0	90	
	LE	RG	24.57	20	15.875	5	60	*0.014
		CG	21.85	15	23.611	0	90	
10.000 Hz	RE	RG	24.57	20	16.377	5	75	0.720
		CG	27.43	20	23.338	5	95	
	LE	RG	27.71	20	18.839	0	75	0.059
		CG	25.86	15	26.554	0	95	
11.200 Hz	RE	RG	29.14	25	19.193	0	80	*0.031
		CG	25.00	15	25.205	0	95	
	LE	RG	32.57	30	18.879	5	80	*0.010
		CG	26.43	20	26.250	0	95	
12.500 Hz	RE	RG	24.85	25	17.024	-5	65	0.893
		CG	29.09	20	23.567	5	95	
	LE	RG	31.47	30	22.547	0	80	0.571
		CG	30.00	25	21.506	0	85	
14.000 Hz	RE	RG	28.44	27.5	20.495	-10	65	0.806
		CG	24.14	20	17.982	5	65	
	LE	RG	30.67	35	21.685	-10	65	0.282
		CG	27.00	25	18.828	0	60	
16.000 Hz	RE	RG	25.23	27.5	19.849	-5	50	0.361
		CG	26.85	20	20.482	0	60	
	LE	RG	25.65	30	18.359	-5	55	0.513
		CG	29.42	27.5	19.043	0	55	

Mann-Whitney test

* p < 0.05 (significant p-value)

**Caption:** EAR= laterality of the ear, Hz = Hertz, FREQ = frequency, SD = standard deviation, MIN = minimum, MAX = maximum

Table 1 indicates the characterization and comparison of the auditory thresholds, as well as of the pure-tone audiometry in conventional frequencies of the research and control groups, associated to the right (RE) and left (LE) ears. Notably, there is a difference between the groups among the frequencies of 250 Hz RE and LE, 500 Hz RE and LE, 2000 Hz RE and 3000 Hz RE, 4000 Hz RE and LE, 6000 Hz RE and LE, and 8000 Hz LE, where the research group presented higher tone means.

Table 2 depicts the comparison between the auditory thresholds of the research group and the control group, considering age and sex. The multiple linear regression model allows the observation of a significant relationship among the audiometric thresholds in frequencies from 250 to 8000 Hz (dependent variable) with the variables group, age, and sex, except in frequencies of 500, 1000, 2000 and 6000 Hz Bilateral for sex. This means that hearing thresholds may be related to group, age, and sex.

Table 3 refers to the characterization and comparison of the auditory thresholds in the high-frequency audiometry of the research and control groups’ participants, associated with the right and left ears. It is possible to observe, in both ears, a difference between the mean thresholds of the research and control groups in the frequencies of 9000 Hz and 11,200 Hz, furthermore, the research group also presented higher pure-tone means when compared to the control group.

Table 4 demonstrates the comparison between the research and control groups’ results from the contralateral and ipsilateral acoustic reflex assessment of the right and left ears, according to the frequency and analyzed as present or absent. The research group showed a higher occurrence of absent acoustic reflexes when compared to the control group, for the frequencies of 500, 1000, 2000, and 4000 Hz in the right ear and the frequencies of 500 and 2000 Hz in the left ear.

**Table 4:** Comparison of the research (RG) and control (GC) groups’ contralateral and ipsilateral acoustic reflex results of the right and left ears

**GROUPS**	**FREQUENCY/EAR**	**RESULT**		**P-VALUE**
		**ABSENT**	**PRESENT**	
RG	**C500 Hz RE**	9	60	* **0.014**
CG		1	70	
RG	**C500 Hz LE**	9	60	*0.048
CG		2	69	
RG	**I500 Hz RE**	3	16	0.066
CG		1	70	
RG	**I500 Hz LE**	3	16	0.066
CG		1	70	
RG	**C1000 Hz RE**	10	59	*0.007
CG		1	70	
RG	**C1000 Hz LE**	8	61	0.085
CG		2	69	
RG	**I1000 Hz RE**	8	61	*0.028
CG		1	70	
RG	**I1000 Hz LE**	11	58	*0.003
CG		1	70	
RG	**C2000 Hz RE**	13	57	*< 0.01
CG		1	70	
RG	**C2000 Hz LE**	10	60	*0.028
CG		2	69	
RG	**I2000 Hz RE**	10	59	*0.007
CG		1	70	
RG	**I2000 Hz LE**	11	58	*0.003
CG		1	70	
RG	**C4000 Hz RE**	29	40	*< 0.01
CG		11	60	
RG	**C4000 Hz LE**	20	49	0.056
CG		10	60	
RG	**I4000 Hz RE**	5	13	0.487
CG		12	59	
RG	**I4000 Hz LE**	4	15	0.802
CG		14	55	

G test

* p < 0.05 (significant p-value)

**Caption:** Hz = Hertz, EAR=laterality of the ear, RE = right ear, LE = left ear, C = contralateral reflex, I = ipsilateral reflex

Table 5 exhibits the comparison between the studied groups of the signal-to-noise ratio (S/N) of T-OAE and DP-OAE by frequency bands and by ear. Comparing the research and control groups’ T-OAE S/N ratio by frequency and by ear, there is a noticeable difference between the means of the two groups for the RE at frequencies of 1000 and 1400 Hz, with the research group presenting a lower mean than the control group. As for the DP-OAE, the difference between the means obtained in the research and control groups was observed for the right ear at frequencies of 1001, 1587, 2002 Hz, 4004 Hz and for the left ear at frequencies of 1587 Hz and 2002 Hz. Like the T-OAE, the lowest means were obtained in the research group.

**Table 5:** Comparison between the S/N ratio by the T-OAE and DP-OAE frequency ranges of the control group (CG) and the research group (RG) (N = 48)

**EAR / FREQUENCY / OAE**	**CG**			**RG**			**P-VALUE**
	**N**	**MEAN**	**STANDARD DEVIATION**	**N**	**MEAN**	**STANDARD DEVIATION**	
**T-OAE**							
RE 1000 Hz	24	11.19	4.27	24	7.21	6.81	* **0.0193**
RE 1400 Hz	24	12.96	4.52	24	9.12	5.26	*0.0094
RE 2000 Hz	24	8.97	3.58	24	8.17	3.39	0.4262
RE 2800 Hz	24	4.74	2.93	24	5.93	4.96	0.3159
RE 4000 Hz	24	0.90	3.23	24	3.20	5.35	0.0785
LE 1000 Hz	24	8.98	6.56	24	3.81	6.63	0.1284
LE 1400 Hz	24	10.28	8.96	24	3.09	5.05	0.2803
LE 2000 Hz	24	7.85	7.41	24	3.35	4.87	0.7159
LE 2800 Hz	24	2.67	5.20	24	4.44	7.88	0.1767
LE 4000 Hz	24	0.84	3.30	24	5.04	6.58	0.1523
**DP-OAE**							
RE 1001 Hz	24	14.97	7.74	24	8.33	7.05	*0.0032
RE 1587 Hz	24	18.34	8.02	24	12.92	7.88	*0.0225
RE 2002 Hz	24	18.06	8.36	24	12.60	6.27	*0.0137
RE 3174 Hz	24	14.37	9.80	24	10.60	6.27	0.1196
RE 4004 Hz	24	17.42	9.00	24	10.66	7.88	*0.0081
RE 6348 Hz	24	9.28	9.63	24	5.99	10.13	0.2559
RE 7996 Hz	24	4.57	9.57	24	0.70	13.03	0.2462
LE 1001 Hz	24	12.96	10.72	24	7.77	5.81	0.2624
LE 1587 Hz	24	17.45	12.99	24	8.71	5.83	*0.0427
LE 2002 Hz	24	17.19	11.95	24	9.95	5.97	*0.0320
LE 3174 Hz	24	14.18	10.82	24	8.72	7.50	0.1594
LE 4004 Hz	24	13.55	11.71	24	11.94	7.65	0.5277
LE 6348 Hz	24	7.20	5.64	24	10.48	11.27	0.6213
LE 7996 Hz	24	3.41	2.24	24	10.51	9.97	0.6930

Student's t-test

* p-value < 0.05 (significant p-value)

**Caption:** N = sample size, Hz = Hertz, RE = right ear, LE = left ear, T-OAE = transient stimulus otoacoustic emissions, DP-OAE = distortion product otoacoustic emissions, CG=control group, RG=research group

**Figure 1:**
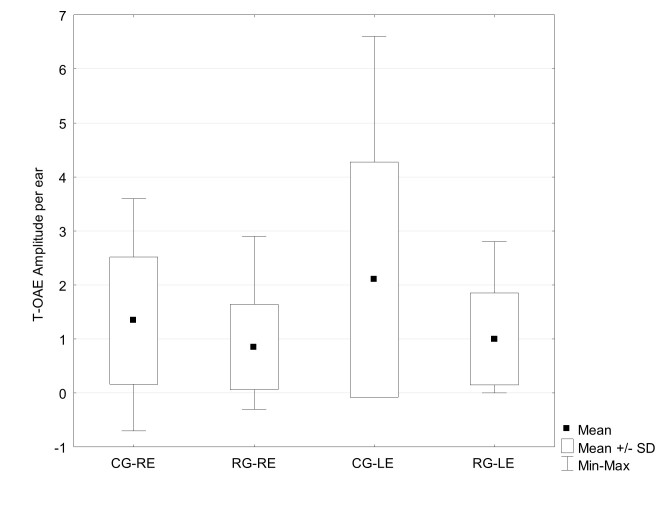
Comparison of the total T-OAE suppression effect in the research (RG) and control (CG) groups associated with the right (RE) and left (LE)

Figure 1 shows the comparison of the total T-OAE suppression effect per ear (right and left) of the research and control groups. The result reveal lower means of the T-OAE suppression effect in the research group and, when using the using Student's t-test to compare the means between the groups, the result indicates a p-value of 0.1171 for the right ear and 0.0450 for the left ear, demonstrating a significant difference between the groups for the left ear.

## DISCUSSION

The results of the present study allowed the analysis of the possible differences among the hearing of family farmers in comparison with the population not exposed to pesticides, as evidenced in the audiological tests.

In the assessment of pure-tone auditory thresholds in conventional frequencies, the results revealed statistical differences between the research and control groups, with the research group showing higher means (Table 1), which corroborates with other studies^(^ 19,20 ^)^.

There is also a significant relationship between tone auditory thresholds according to group, age, and sex (Table 2). The variables research group, males and advanced age were associated with higher audiometric thresholds than the others. Based on this analysis, it can be inferred that the hearing thresholds are influenced by group, age and sex.

In the high-frequency audiometry assessment, statistical differences were observed for the frequencies of 9000 and 11,200 Hz in the right and left ears (Table 3). The audiometric notch in these frequencies may indicate a cochlear alteration in the basal region of the cochlea, and this finding may be useful for the early diagnosis of hearing impairment induced by pesticides, in line with the results reported in another study^(^ 21 ^)^ that evaluated the high frequencies in individuals exposed to further ototoxic agents (noise and solvents).

Another finding of the present study is related to the presence/absence of the contralateral and ipsilateral acoustic reflex assessment in both the research and control groups (Table 4). There was a statistical difference in some frequencies of the right and left ipsilateral and contralateral reflexes. Similar results were observed in a study carried out with tobacco growers exposed to pesticides, which showed significant differences in the contralateral acoustic reflex only at the frequency of 4000 Hz^(^ 4 ^)^.

The absence of suppression of the acoustic reflex could be related to an alteration in the medial olivocochlear efferent auditory system, more specifically in the superior olivary complex, which could have been caused by agrochemical exposure^(^ 22 ^)^.

Regarding the findings of the T-OAE and the DP-OAE, a statistical difference was observed between the means of the two groups, with lower responses in the research group (Table 5). The same result was observed in other studies^(^ 5 ^)^, suggesting that agrochemical exposure increases the risk of damage to the cochlear function.

When analyzing the results of the T-OAE suppression effect between the research and control groups (Figure 1), minor effects were shown in the research group, which was also observed by another research that studied the suppression effect in workers exposed to pesticides^(^ 5 ^)^. This finding may refer to the fact that pesticides reduce the inhibitory effect of the medial olivocochlear efferent system, which is responsible for adjusting the active process of the cochlea, attenuating rapid contractions through neurotransmitters. However, this hypothesis must be interpreted with caution, since significant results were observed only for the left ear in the present study.

The tests used herein were selected due to their availability in the medium-complexity services of the SUS (Portuguese acronym for Sistema Único de Saúde - Unified Health System, Brazil's publicly funded health care), and because they were recommended to assess the effects of pesticides^(^ 14 ^)^. With that being said, each service can structure its audiological evaluation protocol, requiring the inclusion of a minimum amount of tests to assess the extent of auditory damage, since there is no consensus on the appropriate protocol regarding ototoxic chemical agents^(^ 14 ^)^.

Despite the evidence of risk of damage induced by agrochemical exposure to both auditory and general health described in the literature^(^ 3-14,21,23-28 ^)^, government officials, health professionals and workers are either unaware of or ignore this issue.

Therefore, the National Guidelines for Health Surveillance of Populations Exposed to Pesticides^(^ 29 ^)^, and the Protocol for the Assessment of Chronic by Pesticide Poisoning^(^ 14 ^)^, were created in Brazil to guide SUS’s healthcare network in the diagnosis, treatment, rehabilitation, promotion, prevention, and surveillance of workers exposed to pesticides, and should be used to identify, assess or monitor the general and auditory health of agricultural workers exposed to pesticides.

Actions that help promote the proper use of pesticides are recommended, as well as the dissemination of other cultivation methods that do not involve contact with pesticides. Actions to promote hearing health and to prevent hearing loss are essential for family farmers, and it should be emphasized that these actions must be collectively developed at all levels of health care, with government officials and workers aiming at improving the agricultural workers’ quality of life.

### Study limitations

An important limitation of the present study was the lack of quantitative data on agrochemical exposure, which limited the dose/response analysis. Therefore, it is not possible to determine safe levels of agrochemical exposure for hearing health, nor is it possible to reach a conclusion regarding the cause/effect relationship, only that there is an association between agrochemical exposure and peripheral hearing disorders. The sample size of both the research and the control groups may have influenced the statistical analysis of the T-OAE suppression effect results. Finally, this study did not use an exhaustive set of tests, as suggested by some authors.

## CONCLUSION

Differences were found among the hearing of family farmers when compared to the population not exposed to pesticides. Conventional auditory thresholds were associated with exposure, age, and sex. It was also observed that agricultural workers exposed to pesticides may present hearing impairments, characterized by the absence of stapedial reflex, reduction in the T-OAE signal-to-noise ratio and dysfunction of the olivocochlear efferent auditory system.
