# Supplementary material for: Audiological findings of family farmers exposed to pesticides
Source: Codas. 2023 Sep 1;35(4):e20220108. doi: 10.1590/2317-1782/20232022108en (PMC10546921; doi:10.1590/2317-1782/20232022108en)
Supplement: Translation

# Translation: Achados audiológicos de agricultores familiares paranaenses expostos a agrotóxicos

**DOI:** 10.1590/2317-1782/20232022108pt

**Authors:** Diolen Conceição Barros Lobato, Patrícia Arruda de Souza Alcarás, Denise Maria Vaz Romano França, Cláudia Giglio de Oliveira Gonçalves, Adrian Fuente, Adriana Bender Moreira de Lacerda

**Affiliations:** 1 Programa de Pós-graduação “Mestrado e Doutorado” em Distúrbios da Comunicação, Universidade Tuiuti do Paraná - UTP - Curitiba (PR), Brasil.; 2 Departamento de Educação, Universidade Estadual do Paraná - UNESPAR - Curitiba (PR), Brasil.; 3 École d´Orthophonie et d´Audiologie, Université de Montréal - UdeM - Montréal (Québec), Canadá.

**Keywords:** Agrotóxicos, Audição, Perda Auditiva, Riscos Ocupacionais, Saúde do Trabalhador

## Abstract

**Objetivo:**

Analisar as possíveis diferenças na audição de agricultores e suas famílias em comparação com população não exposta aos agrotóxicos.

**Método:**

Estudo transversal, com 70 participantes (grupo pesquisa), com média de idade de 39,7 anos, de ambos os sexos, proprietários de pequenos estabelecimentos agrícolas vinculados a agricultura familiar e tempo de exposição médio aos agrotóxicos de 23,7 anos. Um grupo controle com 71 participantes de ambos os sexos, sem exposição a ruído e agentes químicos, com idade média de 39,5 anos, foi incluído para a comparação dos resultados. Na etapa 1, ambos os grupos foram submetidos a audiometria convencional e de altas frequências e imitanciometria. Na etapa 2, somente os normoouvintes foram submetidos as emissões otoacústicas evocadas e efeito de supressão das emissões otoacústicas transientes.

**Resultados:**

Observou-se diferenças significativas entre os grupos para audiometria tonal convencional e de altas frequências, e no reflexo acústico. As frequências mais afetadas na audiometria tonal convencional foram 3 a 6kHz e na audiometria de altas frequências foram as frequências de 9.000 e 11.200 Hz. Nas emissões otoacusticas transientes, observou-se no efeito de supressão piores resultados no grupo de pesquisa.

**Conclusão:**

Conclui-se que houve diferenças na audição dos agricultores familiares em comparação com o grupo controle. Os limiares auditivos convencionais estão relacionados em função do grupo, idade e gênero. O trabalho na agricultura está associado com o comprometimento na região basal da cóclea, à ausência de reflexo acústico, à redução da relação sinal/ruído das emissões otoacústicas transientes e à disfunção do sistema auditivo eferente olivococlear.

## INTRODUÇÃO

O agricultor familiar^(^ 1 ^)^ é um trabalhador que desempenha suas funções na área rural e apesar da existência de políticas públicas voltadas à Saúde do Trabalhador, em muitas situações desconhece os riscos do seu ambiente de trabalho à sua saúde e de seus familiares, bem como os meios para prevenir os danos ou os efeitos relacionados ao trabalho.

Dentre os riscos de trabalho do agricultor familiar, destaca-se a exposição aos agrotóxicos, que são substâncias químicas utilizadas na agricultura com o intuito de alterar a composição da flora ou da fauna, visando combater as ações danosas de seres nocivos vivos presentes no meio ambiente. No entanto, a sua utilização não altera somente a composição do meio ambiente, mas gera inúmeros efeitos deletérios à saúde^(^ 2-4 ^)^.

Na saúde auditiva, estudos mostram efeitos nocivos no sistema auditivo periférico^(^ 3,5-7 ^)^ e central^(^ 3,8-11 ^)^, bem como efeitos danosos no sistema vestibular^(^ 6,12,13 ^)^. Os danos podem ser observados na motilidade das células ciliadas externas do órgão de Corti, progredindo para células ciliadas interna, nervo auditivo, tronco encefálico e regiões corticais, enquanto que no sistema auditivo vestibular, autor aponta lesões nas células ciliadas da crista da ampola do sáculo e do utrículo^(^ 3 ^)^.

Faz-se necessário o monitoramento audiológico e a inclusão dos trabalhadores nos Programas de Conservação Auditiva (PCA)^(^ 2,7,9,11,12 ^)^, pois há evidências de que o dano auditivo pode estar relacionado com a intoxicação endógena por agrotóxicos^(^ 4,14 ^)^ podendo ser considerada uma manifestação precoce de intoxicação crônica por este agente químico^(^ 4,14 ^)^. Porém, os estudos que investigaram a audição de trabalhadores expostos aos agrotóxicos, são compostos por participantes do sexo masculino, na sua grande maioria. Já os estudos com participantes do sexo feminino ou que os participantes foram constituídos por famílias de agricultores rurais (homens, mulheres e filhos) expostos a agrotóxico são escassos ou nulos.

No contexto apresentado, este estudo justifica-se pela magnitude das famílias expostas aos agrotóxicos, dada a relevância da agricultura brasileira como fonte de geração de renda às famílias, principalmente para alguns municípios onde a agricultura desempenha importante papel em regiões com poucas alternativas econômicas, em alguns casos a única oportunidade de renda e/ou emprego para certos grupos de indivíduos.

Sendo assim, o objetivo desse estudo foi analisar as possíveis diferenças na audição de agricultores e suas famílias, compostos por integrantes de ambos os sexos, proprietários de pequenos estabelecimentos agrícolas vinculados à agricultura familiar com exposição aos agrotóxicos em comparação com população não exposta aos agrotóxicos.

## MÉTODO

Este estudo foi aprovado pelo Comitê de Ética em Pesquisa em Humano e Animais, da Universidade Estadual do Centro Oeste do Paraná, COMEP/UNICENTRO Ofício nº 081/2011, folha de rosto nº 413146 e parecer 023/2011, com data de 17 de outubro de 2011. Trata-se de um estudo transversal, desenvolvido no estado do Paraná (Brasil), com agricultores familiares pertencentes às cidades do interior. Todos os participantes foram incluídos no estudo mediante assinatura no Termo de Consentimento Livre e Esclarecido.

As avaliações foram realizadas pelo mesmo profissional, porém em três clínicas universitárias localizadas no estado do Paraná\ Brasil. Declara-se que em todas as clínicas que ocorreram a coleta dos dados os procedimentos foram realizados com os mesmos critérios e cuidados, e todos os testes que compuseram a avaliação audiológica foram realizados no mesmo período. Informa-se que a audiometria e as emissões otoacústicas foram realizadas em cabinas acústicas, aferidas anualmente de acordo com a norma de ruído estabelecida pela ANSI S3.1^(^ 15 ^)^.

O presente estudo foi realizado em duas etapas:

Etapa 1

O recrutamento dos participantes foi realizado pela Secretaria de Saúde do Estado do Paraná, por meio de busca ativa de pelo menos um caso de intoxicação por agrotóxico na família, nos municípios selecionados. Os participantes foram convidados pelos agentes comunitários de saúde dos municípios, por comunicação oral, pessoalmente ou por contato telefônico.

A amostra da etapa 1 foi composta por dois grupos, pesquisa e controle. O grupo de pesquisa foi formado por 70 participantes, proprietários de pequenos estabelecimentos agrícolas vinculados a agricultura familiar e seus familiares, com faixa etária de 18 a 76 anos (Média=39,7 anos; Dp=13,4 anos), com ausência de cerúmen ou corpo estranho no meato acústico externo, doenças crônicas ou otológicas pregressas, com normalidade de orelha média, sem exposição ao ruído ocupacional (auto-relatado), e tempo de exposição médio ao agrotóxico de 23,7 anos (Dp=12,9 anos), sendo o mínimo de exposição de um ano e o máximo de 60 anos, dos quais 26 (37%) são do sexo feminino e 44 (63%) do sexo masculino. O grupo controle foi formado por 71 participantes, sem histórico de exposição a ruído e agentes químicos (auto-relatado), pertencentes ao banco de dados do Serviço de Audiologia de uma Universidade do Paraná, com a faixa etária de 18 a 67 anos (Média=39,5 anos), sendo 27 (38%) do sexo feminino e 44 (62%) do sexo masculino.

Informa-se que o grupo de pesquisa referiu ter contato com diversos tipos de agrotóxicos, como o glifosato (80%), a dinitroanilina (53%), o organofosforado (51%), o piretróide (49%), o neonicotinoide (46%), o ditiocarbamato (43%), o carbamato (17%) e o organoclorado (3%). O manuseio dos agrotóxicos se dá mediante o preparo da calda (57%), aplicação (85%) e lavagem do material (67%). 70% desse grupo relataram aplicar os agrotóxicos por meio da bomba costal, 8% por meio de spray e 15% referiram não utilizar nenhum tipo de equipamento. Durante a aplicação, 41% referiram utilizar botas/sapatos, 31% blusa, 29% luvas, 27% máscara com filtro, 25% calça, 21% macacão, 19% óculos, 12% proteção na cabeça, 4% máscara sem filtro e 4% roupas descartáveis.

Os participantes foram submetidos à meatoscopia através do otoscópio da marca Mikatos, com a finalidade de avaliar o meato acústico externo e observar a presença ou não de corpo estranho, o que impediria a realização adequada da avaliação audiológica. Em seguida, foi realizada a audiometria tonal liminar, com pesquisa dos limiares nas frequências de 250, 500, 1000, 2000, 3000, 4000, 6000 e 8000 Hertz (Hz) por via aérea e 500, 1000, 2000, 3000 e 4000 Hz por via óssea quando a via aérea encontrava-se alterada. Considerou-se limiares dentro da normalidade até 25 dBNA, para todas as frequências, de acordo com o Anexo II, da NR - 7^(^ 16 ^)^. A audiometria foi realizada com dois audiômetros: um da marca Otometrics, modelo Madsen Itera II, com fone supra-aural TDH-39, e outro da marca Damplex, modelo DA 65, com fone supra-aural TDH-39, devidamente calibrados. A audiometria de altas-frequências foi realizada com audiômetro da marca Otometrics, modelo Madsen Itera II, com fone supra-aural HDA-200 e estímulo em dBNA, sendo avaliadas, por via aérea, as frequências de 9000, 10000, 11200, 12500, 14000 e 16000 Hz e os resultados foram comparados com o grupo controle^(^ 17 ^)^_._

Para as medidas de imitância acústica, também foram utilizados dois equipamentos: um da marca Interacoustics, modelo AT22 e outro da marca Audiotest, modelo 425, devidamente calibrados. Fizeram parte das medidas de imitância acústica: a timpanometria e a pesquisa do reflexo acústico, contra e ipsilateral, nas frequências de 500,1000, 2000 e 4000 Hz. Na timpanometria considerou-se como resultado normal valores obtidos na complacência estática entre 0,3 ml a 1,3 ml e pico pressórico de orelha média entre -100 daPa a +100 daPa. Valores abaixo de 0,3 e acima de 1,3 foram considerados alterados, bem como pressões de orelha média menores que -100 daPa e maiores que +100 daPa. Na pesquisa do reflexo acústico foi considerado como presente quando desencadeado na intensidade máxima permitida pelo equipamento (120 dBNA - aferência contralateral) e (110 dBNPS - na aferência ipsilateral) e ausente quando não desencadeado na intensidade máxima permitida pelo equipamento^(^ 18 ^)^.

Etapa 2

Foram incluídos somente os participantes que apresentaram limiares auditivos dentro dos padrões de normalidade^(^ 16 ^)^ ou perda auditiva ≤ 40 dBNA de acordo com a média de 500, 1000 e 2000 Hz^(^ 18 ^)^ ou em frequência isolada (3000, 4000, 6000 ou 8000 Hz) e curva timpanométrica tipo A.

O grupo de pesquisa (n=24) foi constituído por participantes com faixa etária entre 24 a 61 anos (Média=36,8 anos), sendo 14 do sexo feminino e 10 do sexo masculino, com tempo de exposição de risco médio de 20,7 anos (mínimo de exposição dois anos e máximo 46 anos). Já o grupo controle (n=24) foi pareado com o grupo de pesquisa levando em consideração a faixa etária e o sexo, e constituído por participantes sem exposição a ruído e produtos químicos, com idade média de 34,7 anos, variando entre 21 e 53 anos, sendo 14 do sexo feminino e 10 do sexo masculino.

Os participantes de ambos os grupos foram submetidos às Emissões Otoacústicas por Estímulo Transiente (EOA-T), Emissões Otoacústicas por Produto de Distorção (EOA-PD) e Efeito de Supressão das EOA-T.

Para a pesquisa das emissões otoacústicas evocadas (EOA-T, EOA-PD e Efeito de Supressão das EOA-T) utilizou-se o programa de computador ILO-V6 - Otodynamics Analyser, acoplado a um notebook HP. O equipamento possui uma sonda (ILO Type OAE Probe), que tem como função a liberação do estímulo sonoro, a recepção e a mensuração das respostas no conduto auditivo externo. Esta sonda é conectada a dois canais e a uma interface acoplada ao notebook.

No exame de EOA-T utilizou-se o seguinte protocolo: estímulo clique nas intensidades do estímulo de 80 dBNPS, 400 varreduras. Considerou-se o padrão de resposta reprodutibilidade geral da resposta maior que 50%, estabilidade da sonda maior que 70%, nível de resposta maior que o ruído, com relação sinal/ruído ≥ 3dB NPS em pelo menos três ou mais frequências consecutivas^(^ 19 ^)^.

A pesquisa das EOA-PD foi realizada com dois tons primários (f1 e f2), sendo f1 a frequência mais baixa e f2 a frequência mais alta. A distância entre as duas frequências (f1 e f2) foram obtidas em uma razão f2/f1 =1,22, a fim de obter o produto ideal de distorção, no modo “Diagnóstico Geral”, e a intensidade utilizada foi L1= 65 dBNPS e L2= 55 dBNPS. A resposta do produto de distorção foi obtida em 2f1-f2 e reportada na frequência f2, como 1001, 1587, 2020, 3174, 4004, 6348 e 7996 Hz. Considerando-se estabilidade da sonda maior que 70% e relação sinal-ruído ≥ 6 dB NPS por frequência específica^(^ 19 ^)^.

Para analisar o efeito de supressão das EOA-T, os participantes que tiveram presença de emissões otoacústicas transientes, com relação sinal/ruído (S/R) igual ou superior a 3 dBNPS em três frequências consecutivas e em ao menos uma orelha, foram submetidos ao teste do efeito de supressão. Sem que houvesse o reposicionamento da sonda, a supressão das EOA-T foi registrada de acordo com o seguinte protocolo: estímulo clique, linear, com intensidade do estímulo em 60 dB NPS (± 5) e ruído branco contralateral em 60 dB NPS (± 5). Em média, foram feitas 500 varreduras, sendo 250 varreduras na ausência do ruído contralateral e 250 na presença do ruído contralateral. A supressão foi avaliada em cada orelha (direita e esquerda). A constatação do efeito de supressão foi realizada em função do nível de resposta geral na presença de ruído contralateral em relação ao nível de resposta geral sem ruído contralateral, tendo como referência dois tipos de respostas: Supressão presente, quando houve redução do nível de resposta geral das emissões na presença de ruído contralateral (valores maiores ou iguais a 1,0) e Supressão ausente, quando a redução do nível de resposta geral das emissões na presença de ruído contralateral não ocorreu (valores menores a 1,0)^(^ 20 ^)^.

A análise estatística da presente pesquisa foi realizada com a utilização de métodos descritivos e inferenciais. Os métodos descritivos (tabelas de frequências absolutas e relativas, média, mínimo, máximo e desvio padrão) foram utilizados para a caracterização dos agricultores familiares em relação às variáveis: idade, sexo e tempo de exposição ao agrotóxico, sinais, sintomas, riscos, resultados das audiometrias convencionais e altas frequências. Os métodos inferenciais: Teste t de Student (para comparar as amplitudes e relação S/R nas EOA-T e EOA-PD, além do efeito de supressão), Coeficiente de Correlação R de Spearman (para avaliar a correlação entre os testes audiológicos: audiometria tonal liminar, audiometria de altas frequências, EOA-T, EOA-PD e efeito de supressão das EOA-T e os exames laboratoriais. Teste de Fisher e Teste Qui-Quadrado (EOA-T, EOA-PD e efeito de supressão das EOA-T), Teste de Man Whitney (comparar os limiares auditivos dos agricultores familiares na audiometria tonal liminar e audiometria de altas frequências) e Modelo de Regressão Linear múltipla (Variável dependente: limiares audiométricos; variáveis independentes: grupo, idade e sexo), Teste G (pesquisa do reflexo acústico). Todos os testes levaram em consideração o nível de significância de 5% (0,05), ou seja, quando o valor de p for menor ou igual a 0,05 existe significância estatística.

## RESULTADOS

Os resultados da caracterização dos limiares auditivos obtidos através da audiometria tonal liminar convencional e de altas frequências dos grupos de pesquisa e controle encontram-se nas Tabelas 1, 2 e 3.

**Tabela 1:** Caracterização e comparação da audiometria tonal liminar convencional do grupo pesquisa (GP) e grupo controle (GC), obtidos nas orelhas direita (OD) e esquerda (OE) (N=141)

**FREQ**	**ORELHA**	**GRUPO**	**MÉDIA**	**MEDIANA**	**DP**	**MÍN**	**MÁX**	**P-VALOR**
**250Hz**	OD	GP	14,79	15	8,741	5	60	*0,000
		GC	10,00	10	4,053	0	5	
	OE	GP	13,57	15	8,269	-5	50	*0,000
		GC	8,87	0	3,891	0	20	
**500Hz**	OD	GP	13,57	10	6,818	5	40	*0,000
		GC	9,30	10	4,728	0	20	
	OE	GP	12,79	10	7,403	0	45	*0,001
		GC	8,59	10	4,719	0	15	
**1000Hz**	OD	GP	11,43	10	7,231	0	40	0,069
		GC	9,00	10	5,664	0	20	
	OE	GP	11,36	10	8,678	0	50	0,206
		GC	8,95	10	5,664	0	25	
**2000Hz**	OD	GP	13,07	10	12,047	0	60	*0,022
		GC	8,50	10	6,740	0	20	
	OE	GP	12,71	10	13,875	-5	60	0,418
		GC	8,30	10	6,900	0	35	
**3000Hz**	OD	GP	16,07	10	16,127	-5	65	*0,018
		GC	9,37	10	7,316	0	25	
	OE	GP	16,57	10	18,209	-5	70	0,074
		GC	10,21	10	8,760	0	40	
**4000Hz**	OD	GP	18,93	15	17,464	-5	75	*0,017
		GC	11,55	10	8,088	0	35	
	OE	GP	19,93	15	17,703	-5	70	*0,001
		GC	11,70	10	10,452	0	55	
**6000Hz**	OD	GP	22,93	20	16,539	5	85	* **0,000**
		GC	13,00	15	7,995	0	40	
	OE	GP	24,00	20	17,664	-5	90	*0,000
		GC	13,38	10	10,750	0	50	
**8000Hz**	OD	GP	17,93	15	16,495	0	90	0,104
		GC	13,96	10	9,949	0	40	
	OE	GP	20,43	15	18,013	-5	75	*0,016
		GC	14,44	10	14,231	0	65	

Teste de Mann-Whitney

* p<0,05 (valor de p significante)

**Legenda:** Hz = Hertz, FREQ = frequência, DP = desvio padrão, MÍN = mínimo, MÁX = máximo

**Tabela 2:** Análise do modelo de regressão linear múltipla em relação a comparação dos limiares auditivos convencionais conforme os grupos (pesquisa e controle), a idade e o sexo (N=141)

		**250 Hz**	**500 Hz**	**1 kHz**	**2 kHz**	**3 kHz**	**4 kHz**	**6 kHz**	**8 kHz**
**Grupo**	OD	*0,000	*0,000	*0,015	*0,002	*0,000	*0,000	*0,000	*0,007
	OE	*0,000	*0,000	*0,022	*0,028	*0,002	*0,000	*0,000	*0,007
**Idade**	OD	*0,000	*0,000	*0,000	*0,000	*0,000	*0,000	*0,000	*0,000
	OE	*0,000	*0,000	*0,000	* **0,000**	*0,000	*0,000	*0,000	*0,000
**Sexo**	OD	*0,010	>0,05	>0,05	>0,05	*0,005	*0,009	>0,05	*0,010
	OE	*0,000	>0,05	>0,05	>0,05	*0,002	*0,001	>0,05	*0,006

Modelo de regressão linear múltipla

* p<0,05 (valor de p significante)

**Legenda:** Hz = Hertz, OD = orelha direita, OE = orelha esquerda

**Tabela 3:** Caracterização e comparação da audiometria tonal liminar em altas frequências do grupo pesquisa (GP) e grupo controle (GC), obtidos nas orelhas direita (OD) e esquerda (OE) (N=141)

**FREQ**	**ORELHA**	**GRUPO**	**MÉDIA**	**MEDIANA**	**DP**	**MÍN**	**MÁX**	**P-VALOR**
9.000Hz	OD	GP	24,00	15	17,565	0	90	*0,006
		GC	19,29	15	19,821	0	90	
	OE	GP	24,57	20	15,875	5	60	*0,014
		GC	21,85	15	23,611	0	90	
10.000Hz	OD	GP	24,57	20	16,377	5	75	0,720
		GC	27,43	20	23,338	5	95	
	OE	GP	27,71	20	18,839	0	75	0,059
		GC	25,86	15	26,554	0	95	
11.200Hz	OD	GP	29,14	25	19,193	0	80	*0,031
		GC	25,00	15	25,205	0	95	
	OE	GP	32,57	30	18,879	5	80	* **0,010**
		GC	26,43	20	26,250	0	95	
12.500Hz	OD	GP	24,85	25	17,024	-5	65	0,893
		GC	29,09	20	23,567	5	95	
	OE	GP	31,47	30	22,547	0	80	0,571
		GC	30,00	25	21,506	0	85	
14.000Hz	OD	GP	28,44	27,5	20,495	-10	65	0,806
		GC	24,14	20	17,982	5	65	
	OE	GP	30,67	35	21,685	-10	65	0,282
		GC	27,00	25	18,828	0	60	
16.000Hz	OD	GP	25,23	27,5	19,849	-5	50	0,361
		GC	26,85	20	20,482	0	60	
	OE	GP	25,65	30	18,359	-5	55	0,513
		GC	29,42	27,5	19,043	0	55	

Teste de Mann-Whitney

* p<0,05 (valor de p significante)

**Legenda:** Hz = Hertz, FREQ = frequência, DP = desvio padrão, MÍN = mínimo, MÁX = máximo

Na Tabela 1 observa-se a caracterização e comparação dos limiares auditivos, da audiometria tonal liminar nas frequências convencionais dos grupos de pesquisa e controle relacionados às orelhas direita (OD) e esquerda (OE). Nota-se haver diferença entre os grupos nas frequências de 250 Hz OD e OE, 500 Hz OD e OE, 2000 Hz OD, 3000 Hz OD, 4000 Hz OD e OE, 6000 Hz OD e OE e 8000 Hz OE, sendo que o grupo pesquisa apresentou maiores médias tonais.

A Tabela 2 apresenta a comparação dos limiares auditivos do grupo de pesquisa com o grupo controle, a idade e o sexo. Através do modelo de regressão linear múltipla, verifica-se que existe uma relação significativa entre os limiares audiométricos nas frequências de 250 a 8000 Hz (variável dependente) com as variáveis grupo, idade e sexo, exceto nas frequências de 500, 1000, 2000 e 6000 Hz bilateral para o sexo. Isso significa que os limiares auditivos podem estar relacionados em função do grupo, idade e sexo.

A Tabela 3 refere-se à caracterização e comparação dos limiares auditivos na audiometria de altas frequências, dos participantes dos grupos de pesquisa e controle, relacionados às orelhas direita e esquerda. É possível observar, em ambas orelhas, diferença entre as médias dos limiares dos grupos pesquisa e controle nas frequências de 9000 Hz e 11.200 Hz, sendo que o grupo pesquisa também apresentou maiores médias tonais em comparação com o grupo controle.

A Tabela 4 apresenta a comparação dos resultados da pesquisa do reflexo acústico contralateral e ipsilateral das orelhas direita e esquerda, dos grupos pesquisa e controle, em função da frequência e analisados como presentes ou ausentes. O grupo pesquisa apresentou maior ocorrência de reflexos acústicos ausentes em comparação com o grupo controle, para as frequências de 500, 1000, 2000 e 4000 Hz em orelha direita e para as frequências de 500 e 2000 Hz em orelha esquerda.

**Tabela 4:** Comparação dos resultados dos grupos de pesquisa (GP) e controle (GC) do reflexo acústico contralateral e ipsilateral das orelhas direita e esquerda

**GRUPOS**	**FREQUÊNCIA/ORELHA**	**RESULTADO**		**P-VALOR**
		**AUSENTE**	**PRESENTE**	
GP	**C500HzOD**	9	60	*0,014
GC		1	70	
GP	**C500HzOE**	9	60	*0,048
GC		2	69	
GP	**I500HzOD**	3	16	0,066
GC		1	70	
GP	**I500HzOE**	3	16	0,066
GC		1	70	
GP	**C1000HzOD**	10	59	*0,007
GC		1	70	
GP	**C1000HzOE**	8	61	0,085
GC		2	69	
GP	**I1000Hz OD**	8	61	*0,028
GC		1	70	
GP	**I1000Hz OE**	11	58	*0,003
GC		1	70	
GP	**C2000HzOD**	13	57	*< 0,01
GC		1	70	
GP	**C2000HzOE**	10	60	*0,028
GC		2	69	
GP	**I2000HzOD**	10	59	*0,007
GC		1	70	
GP	**I2000HzOE**	11	58	*0,003
CG		1	70	
GP	**C4000HzOD**	29	40	*< 0,01
GC		11	60	
GP	**C4000HzOE**	20	49	0,056
GC		10	60	
GP	**I4000HzOD**	5	13	0,487
GC		12	59	
GP	**I4000HzOE**	4	15	0,802
GC		14	55	

Teste G

* p<0,05 (valor de p significante)

**Legenda:** Hz = Hertz, OD = orelha direita, OE = orelha esquerda, C = reflexo contralateral, I = reflexo ipsilateral

Na Tabela 5 é apresentada a comparação da relação sinal/ruído (S/R) das EOA-T e das EOA-PD por bandas de frequência e por orelha, entre os grupos estudados. Comparando a relação S/R das EOA-T por frequência e por orelha entre os grupos pesquisa e controle, verifica-se que existe diferença entre as médias dos dois grupos para a OD nas frequências de 1000 e 1400 Hz, sendo o grupo pesquisa com menores médias em relação ao grupo controle. Já nas EOA-PD, a diferença entre as médias obtidas nos grupos pesquisa e controle foi observada para a orelha direita nas frequências de 1001, 1587, 2002 Hz, 4004 Hz e para a orelha esquerda nas frequências de 1587 Hz e 2002 Hz. Igualmente as EOA-T, as menores médias foram obtidas no grupo pesquisa.

**Tabela 5:** Comparação da relação S/R por faixa de frequência das EOA-T e das EOA-PD, entre o grupo controle (GC) e o grupo pesquisa (GP) (N=48).

**ORELHA / FREQUÊNCIA / EOA**	**GC**			**GP**			**P-VALOR**
	N	**MÉDIA**	**DESVIO PADRÃO**	**N**	**MÉDIA**	**DESVIO PADRÃO**	
**EOA-T**							
OD 1000 Hz	24	11,19	4,27	24	7,21	6,81	*0,0193
OD 1400 Hz	24	12,96	4,52	24	9,12	5,26	*0,0094
OD 2000 Hz	24	8,97	3,58	24	8,17	3,39	0,4262
OD 2800 Hz	24	4,74	2,93	24	5,93	4,96	0,3159
OD 4000 Hz	24	0,90	3,23	24	3,20	5,35	0,0785
OE 1000 Hz	24	8,98	6,56	24	3,81	6,63	0,1284
OE 1400 Hz	24	10,28	8,96	24	3,09	5,05	0,2803
OE 2000 Hz	24	7,85	7,41	24	3,35	4,87	0,7159
OE 2800 Hz	24	2,67	5,20	24	4,44	7,88	0,1767
OE 4000 Hz	24	0,84	3,30	24	5,04	6,58	0,1523
**EOA-PD**							
OD 1001 Hz	24	14,97	7,74	24	8,33	7,05	* **0,0032**
OD 1587 Hz	24	18,34	8,02	24	12,92	7,88	*0,0225
OD 2002 Hz	24	18,06	8,36	24	12,60	6,27	*0,0137
OD 3174 Hz	24	14,37	9,80	24	10,60	6,27	0,1196
OD 4004 Hz	24	17,42	9,00	24	10,66	7,88	*0,0081
OD 6348 Hz	24	9,28	9,63	24	5,99	10,13	0,2559
OD 7996 Hz	24	4,57	9,57	24	0,70	13,03	0,2462
OE 1001 Hz	24	12,96	10,72	24	7,77	5,81	0,2624
OE 1587 Hz	24	17,45	12,99	24	8,71	5,83	*0,0427
OE 2002 Hz	24	17,19	11,95	24	9,95	5,97	*0,0320
OE 3174 Hz	24	14,18	10,82	24	8,72	7,50	0,1594
OE 4004 Hz	24	13,55	11,71	24	11,94	7,65	0,5277
OE 6348 Hz	24	7,20	5,64	24	10,48	11,27	0,6213
OE 7996 Hz	24	3,41	2,24	24	10,51	9,97	0,6930

Teste T de Student

* p valor<0,05 (valor de p significante)

**Legenda:** N = tamanho da amostra, Hz = Hertz, OD = orelha direita, OE = orelha esquerda, EOA-T = emissões otoacústicas por estímulo transiente, EOA-PD = emissões otoacústicas por produto de distorção

A Figura 1 demonstra a comparação do efeito de supressão total da EOA-T dos grupos pesquisa e controle em função das orelhas (direita e esquerda). O resultado evidencia menores médias do efeito de supressão das EOA-T no grupo pesquisa e, ao comparar as médias entre os grupos, por meio do teste estatístico *t* de Student, o resultado evidenciou valor de p = 0,1171 para a orelha direita e p = 0,0450 para a orelha esquerda, demonstrando diferença significante entre os grupos para a orelha esquerda.

**Figura 1:** Comparação do efeito de supressão total das EOA-T dos grupos pesquisa (GP) e controle (GC) em função das orelhas direita (OD) e esquerda (OE)

## DISCUSSÃO

Os resultados do presente estudo permitiram analisar as possíveis diferenças na audição dos agricultores familiares em comparação com a população não exposta aos agrotóxicos, evidenciadas nos testes audiológicos.

Na pesquisa dos limiares auditivos tonais em frequências convencionais, os resultados revelaram diferenças estatísticas entre os grupos pesquisa e controle, sendo o grupo pesquisa com médias mais elevadas (Tabela 1), corroborando com outros estudos^(^ 19,20 ^)^.

Existe também relação significativa dos limiares auditivos tonais de acordo com o grupo, a idade e o sexo (Tabela 2). O grupo de pesquisa, o sexo masculino e a idade avançada apresentaram limiares audiométricos mais elevados que os demais. A partir dessa análise, sabe-se que os limiares auditivos são influenciados pelo grupo, pela idade e pelo sexo.

Na análise da audiometria de altas frequências observam-se diferenças estatísticas para as frequências de 9000 e 11.200Hz nas orelhas direita e esquerda (Tabela 3). O entalhe audiométrico nessas frequências pode indicar alteração coclear na região basal da cóclea, e este achado pode ser útil no diagnóstico precoce do dano auditivo induzido por agrotóxicos, assim como relatado em outro estudo^(^ 21 ^)^ que avaliou as altas frequências em indivíduos expostos a outros agentes otoagressores (ruído e solventes).

Outro achado do estudo relaciona-se aos resultados referentes à presença/ausência da pesquisa do reflexo acústico contralateral e ipsilateral dos grupos de pesquisa e controle (Tabela 4). Observou-se diferença estatística em algumas frequências do reflexo ipsi e contralateral direito e esquerdo. Resultados semelhantes foram observados em estudo realizado com fumicultores expostos a agrotóxicos o qual evidenciou diferenças significativas no reflexo acústico contralateral, apenas na frequência de 4000Hz^(^ 4 ^)^.

Observa-se que a ausência da supressão do reflexo acústico poderia estar relacionada com uma alteração no sistema auditivo eferente olivococlear medial, mais especificamente no complexo olivar superior, podendo ter sido causada pela exposição a agrotóxicos^(^ 22 ^)^.

Em relação aos achados das EOA-T e EOA-PD, foi observado diferença estatística entre as médias dos dois grupos, com menores respostas no grupo pesquisa (Tabela 5). O mesmo resultado foi observado em outro estudo^(^ 5 ^)^ e, esse achado, sugere que a exposição a agrotóxicos aumenta o risco de danos na função coclear.

Ao analisar os resultados do efeito de supressão das EOA-T entre os grupos de pesquisa e controle (Figura 1), observa-se menores efeitos no grupo pesquisa, dado também observado em outro estudo que estudou o efeito de supressão em trabalhadores expostos a agrotóxicos^(^ 5 ^)^. Esse achado pode remeter ao fato dos agrotóxicos reduzirem o efeito inibidor do sistema eferente olivococlear medial, sendo este sistema responsável pelo ajuste do processo ativo da cóclea, atenuando as contrações rápidas por meio de neurotransmissores. Entretanto essa hipótese deve ser interpretada com cautela, uma vez que nesse estudo foram observados resultados significativos apenas para a orelha esquerda.

Quanto aos testes usados no presente estudo, eles foram selecionados por estarem disponíveis nos serviços de média complexidade do SUS e por terem sido recomendados para avaliar os efeitos dos agrotóxicos^(^ 14 ^)^. Mas, cada serviço pode estruturar o seu protocolo de avaliação audiológica, sendo necessário a inclusão de uma bateria mínima de testes para avaliar a extensão do dano auditivo, uma vez que não existe um consenso sobre o protocolo apropriado para agentes químicos ototóxicos^(^ 14 ^)^.

Apesar da evidência do risco dos danos decorrentes da exposição aos agrotóxicos na saúde auditiva e na saúde geral descritos na literatura^(^ 3-14,21,23-28 ^)^ governantes, profissionais da saúde e trabalhadores desconhecem ou ignoram essa questão.

Para tal, no Brasil, as Diretrizes nacionais para a vigilância em saúde de populações expostas a agrotóxicos^(^ 29 ^)^,e o Protocolo de Avaliação das Intoxicações Crônicas por Agrotóxicos^(^ 14 ^)^, foram criados com a finalidade de orientar a rede de atenção à saúde do SUS no diagnóstico, tratamento, reabilitação, promoção, prevenção e vigilância dos trabalhadores expostos a agrotóxicos, e devem ser usados para identificar, avaliar ou monitorar a saúde geral e auditiva dos trabalhadores agrícolas expostos a agrotóxicos.

Recomenda-se ações que auxiliem e promovam o uso adequado dos agrotóxicos, assim como sejam divulgadas outras formas de cultivo que não necessitem o contato com os agrotóxicos. São imprescindíveis ações de promoção de saúde auditiva e prevenção da perda auditiva para os agricultores familiares, ressaltando-se que essas ações precisam ser trabalhadas em conjunto em todos os níveis de atenção à saúde, governantes e trabalhadores visando melhorias na qualidade de vida do trabalhador agrícola.

### Limitações do estudo

Uma importante limitação para este estudo foi a ausência de dados quantitativos sobre a exposição aos agrotóxicos, o que limitou a análise de relação dose/resposta. Assim, não é possível determinar níveis seguros de exposição aos agrotóxicos para a saúde auditiva, bem como não é possível concluir uma relação causa/efeito, apenas que há associação entre a exposição aos agrotóxicos e as alterações auditivas periféricas. O tamanho da amostra, tanto do grupo de pesquisa quanto do controle, pode ter influenciado na análise estatística dos achados do efeito de supressão das EOA-T. Finalmente, este estudo não utilizou uma bateria de teste exaustiva sugerida por alguns autores.

## CONCLUSÃO

Conclui-se que houve diferenças na audição de agricultores familiares em comparação com população não exposta aos agrotóxicos. Os limiares auditivos convencionais estão relacionados em função da exposição, idade e sexo. Além disso, observou-se que o trabalho na agricultura de pessoas expostas a agrotóxicos pode estar associado à alterações auditivas, caracterizadas por: ausência de reflexo estapediano, redução da relação sinal/ruído das EOA-T e disfunção do sistema auditivo eferente olivococlear.
